# Association between delivery hospitalization blood pressure and severity of postpartum admissions for hypertension

**DOI:** 10.1371/journal.pone.0342836

**Published:** 2026-02-18

**Authors:** Mindy Pike, Judy Han, Leah Chen, Catherine M. Albright, Raj Shree

**Affiliations:** 1 Department of Obstetrics & Gynecology, University of Washington, Seattle, Washington, United States of America; 2 School of Medicine, University of Washington, Seattle, Washington, United States of America; University of North carolina at Greensboro, UNITED STATES OF AMERICA

## Abstract

**Background:**

Hypertension is a leading cause of postpartum admission (PPA), but whether blood pressure (BP) at discharge from delivery hospitalization is associated with PPA severity is unknown. Furthermore, detailed characterization of presentation, management, and progression of hypertensive disorders of pregnancy (HDP) during PPA is lacking.

**Methods:**

Retrospective study of PPAs for hypertension from 2016–2021. Exposure was highest BP within 24 hours of discharge from delivery hospitalization, classified as normal (<140/90 mmHg) or elevated (≥140/90 mmHg). The primary outcome was disease severity, measured by an aggregate “Severity Score” based on severe criteria for HDP. PPAs were further characterized by symptoms, interventions, and HDP progression. We also calculated the proportion of *de novo* postpartum HDP.

**Results:**

Among 132 patients with PPA for hypertension, 76 had normal BP and 56 had elevated BP at discharge. Severity did not differ between groups. Median time to PPA was 5 days (IQR: 3–8) and 3 days (IQR: 1–5) for the normal and elevated BP groups, respectively (β –1.59, 95% CI –3.06, –0.12). Headache was the most frequent presenting symptom. HDP diagnosis worsened in 49% of cases. 27% experienced *de novo* postpartum HDP.

**Conclusions:**

BP at discharge was not associated with PPA severity but was linked to earlier presentation. Close to half had worsening of HDP, while *de novo* hypertension accounted for over a quarter of PPAs. This underscores the need for earlier identification of postpartum hypertensive complications and determination of optimal BP targets. New onset hypertension represents a substantial proportion of PPAs, representing necessary admissions, rather than re-admissions.

## Introduction

Hypertensive disorders of pregnancy (HDP) represent a major contributor to maternal morbidity and mortality [1]. In the United States (US), close to 16% of delivery hospitalizations are complicated by HDP [2]. Two unique HDP phenomena occur in the postpartum period: exacerbation of known HDP and new-onset HDP following a normotensive pregnancy, both of which contribute to postpartum admissions (PPA). Hypertensive complications are a leading cause of PPA [3], and these admissions disrupt typical postpartum recovery, and contribute to substantial costs [4], but, in some cases, may not be preventable.

The American College of Obstetricians and Gynecologists’ (ACOG) 2013 Task Force on Hypertension in Pregnancy recommends treatment of postpartum hypertension when blood pressure (BP) is ≥ 150/100 mmHg [5], while the 2020 ACOG guidelines do not address postpartum BP treatment parameters [1]. Because the American Heart Association recommends diagnosing stage 1 hypertension in non-pregnant adults with SBP ≥ 130 mmHg or DBP ≥ 80 mmHg [6], there is emerging interest in lower BP thresholds for pregnancy and postpartum [7,8].

Contemporary data suggests that 2.5–5.1% of patients with HDP experience PPA for hypertensive complications [4,9–11]. Although these studies have characterized risk factors for PPA for hypertensive complications, they did not characterize the severity of these PPA, the trajectory of worsening HDP from delivery to postpartum, and the proportion of cases that were newly diagnosed postpartum. With studies demonstrating that 90% of PPAs for hypertension occur within 10 days of delivery [9,10], and the lack of clarity regarding postpartum BP targets, we sought to determine differences in HDP severity at PPA and the time to admission based on BP at discharge from delivery hospitalization among individuals admitted for hypertension management postpartum. We also describe the characteristics of presentation and management of PPA for hypertension and the proportion of patients that present with a *de novo* diagnosis in the postpartum period.

## Methods

We conducted a retrospective cohort study of individuals admitted postpartum to our hospital for management of hypertension between 2016–2021 (time period for which data was available). We utilized data from the Obstetrical Care Outcomes Assessment Program (OB COAP), an ongoing, clinician-led, perinatal quality improvement collaborative based in the Northwestern United States that collects clinical data by trained personnel. Quality assurance is performed both at the site and aggregate levels. OB COAP received a determination of exempt status from human subject research review. Additional data abstraction is approved by our university’s Institutional Review Board (STUDY00003732). Data were accessed on 10/25/2023 and authors had access to identifying information during data collection. The requirement for informed consent was waived by the Institutional Review Board. Data were collected and managed using REDCap (Research Electronic Data Capture, Vanderbilt University, Nashville, TN) [12,13].

Patient medical and obstetric history and postpartum interventions were abstracted from the electronic health record for each participant. We excluded those without a documented BP within 24 hours of discharge from delivery hospitalization or if either the delivery or the PPA were not at our hospital. Although our institution does not have a formal protocol for postpartum BP management, the vast majority of providers prescribe labetalol or nifedipine as first line agents. Less commonly prescribed agents include other beta blockers, hydralazine, and angiotensin converting enzyme (ACE) inhibitors, such as enalapril. At the time of delivery hospitalization, we commonly prescribe a 5-day course of furosemide to patients with preeclampsia with severe features, and this is variably done in the setting of PPA for hypertension.

Our exposure was the highest BP within 24 hours of discharge from the delivery hospitalization, with normal (non-exposed) defined as BP < 140/90 mmHg and elevated (exposed) as SBP ≥ 140 mmHg or DBP ≥ 90 mmHg. Our primary outcome was HDP severity at PPA. HDP diagnoses were based on ACOG criteria [1]. To quantify severity, we developed a Severity Score, where 1 point each was assigned for the following: sustained severe BPs (at least two measures of SBP ≥ 160 mmHg or DBP ≥ 110 mmHg at least 15 minutes apart), any lab abnormality (defined as serum creatinine ≥1.1 mg/dL, platelets <100,000/uL, or AST ≥ 76 U/L [twice the upper limit of normal in our clinical lab]), pulmonary edema, PRES (posterior reversible encephalopathy syndrome), and eclampsia. As we had no cases of stroke or cardiac dysfunction, these were not included in the score. Since headache was very frequently present in this cohort and characterization as a “severe” or “non-severe” headache is subjective, we chose to not include this in the Severity Score. We compared the Severity Score between those with elevated versus normal BP at discharge from delivery hospitalization. Our secondary outcomes were days to PPA, defined as days from discharge from delivery hospitalization to PPA for hypertension, and length of PPA.

Linear regression was used to test the association between high BP at delivery hospitalization and Severity Score at PPA. The Severity Score at PPA was described as a categorical variable range (0–5) and treated as a continuous variable in regression analyses. Estimates were adjusted for gestational age at delivery, diagnosis at discharge from delivery hospitalization, whether the patient was discharged on antihypertensives or oral furosemide from delivery hospitalization, length of delivery admission in days (date of delivery to date of discharge), and abnormal lab values at delivery hospitalization. Linear regression was additionally used to test the associations between high BP and time to PPA and length of PPA and adjusted for the same covariates as above except abnormal lab values at delivery hospitalization. We planned one sensitivity analysis using BP ≥ 150/100 (based on ACOG recommendations [5]), and a second using BP ≥ 130/80 mmHg (based on the 2017 AHA/ACC guidelines for the diagnosis of stage 1 hypertension [6]) to define elevated BP at delivery hospitalization.

We also described the clinical characteristics of PPA for hypertension including most frequent presenting symptom and how often the diagnosis worsened from discharge from delivery hospitalization to PPA. For change in diagnosis, we compared the worst HDP diagnosis at discharge from delivery hospitalization to the worst HDP diagnosis at discharge from the PPA, and a worsening of HDP was present in the following situations: a) no HDP diagnosis to any HDP diagnosis; b) CHTN (chronic hypertension) to any preeclampsia phenotype; c) GHTN (gestational hypertension) to any preeclampsia phenotype; d) preeclampsia without severe features to preeclampsia with severe features, HELLP (hemolysis, elevated liver enzymes, low platelets) syndrome, or eclampsia; e) preeclampsia with severe features to HELLP syndrome or eclampsia. Preeclampsia phenotypes included preeclampsia with and without severe features, superimposed preeclampsia, HELLP syndrome, and eclampsia. Those meeting the criteria listed in group “a” above were defined as having *de novo* HDP presenting in the postpartum period. P-values less than 0.05 were considered statistically significant. All analyses were conducted using Stata version 18.0 (StataCorp LLC, College Station, TX).

## Results

Of 11,907 individuals who delivered between 2016 and 2021, 132 met inclusion criteria. 76 were in the normal BP group and 56 in the elevated BP group. Individuals in the elevated BP group were more likely to have delivered at slightly earlier gestational ages, with smaller infants, have a pregnancy at delivery hospitalization complicated by preeclampsia, and have antihypertensives and oral furosemide prescribed at discharge from the delivery hospitalization (Table 1). There were no other demographic differences between groups.

**Table 1:** Patient demographics.

	Total (n = 132)	Normal BP at Discharge from Delivery Hospitalization (<140/90 mmHg) (N = 76)	Elevated BP at Discharge from Delivery Hospitalization (SBP ≥ 140 or DBP ≥ 90 mmHg) (N = 56)
Age > 35 years at delivery	68 (51.5)	38 (50.0)	30 (53.6)
Nulliparous	65 (49.2)	34 (44.7)	31 (55.4)
*Race*			
White	79 (59.9)	36 (47.4)	43 (76.8)
Black	31 (23.5)	25 (32.9)	6 (10.7)
Asian	16 (12.1)	12 (15.8)	4 (7.1)
Other/ Not Reported	6 (4.6)	3 (4.0)	3 (5.4)
Hispanic/Latino Ethnicity	4 (3.0)	2 (2.6)	2 (3.6)
Insurance			
Public	48 (36.4)	33 (43.4)	15 (26.8)
Private	78 (59.1)	40 (52.6)	38 (67.9)
None	6 (4.6)	3 (4.0)	3 (5.4)
BMI ≥ 30 kg/m^2^	84 (63.6)	51 (67.1)	33 (58.9)
*Chronic Medical Conditions*			
None	46 (34.9)	29 (38.2)	17 (30.4)
Chronic Hypertension	27 (20.5)	13 (17.1)	14 (25.0)
Maternal Cardiac Disease	10 (7.6)	5 (6.6)	5 (8.9)
Pregestational Diabetes	5 (3.8)	3 (4.0)	2 (3.6)
Gestational Diabetes	9 (6.8)	6 (7.9)	3 (5.4)
GA at delivery (weeks)	38.4 (37.1, 39.4)	38.9 (37.8, 39.9)	38.1 (36.9, 38.9)
Preterm delivery (<37 weeks GA)	21 (15.9)	7 (9.2)	14 (25.0)
Multiple gestation pregnancy	6 (4.6)	3 (4.0)	3 (5.4)
Cesarean Delivery	72 (54.6)	47 (61.8)	25 (44.6)
Postpartum Hemorrhage	23 (17.4)	16 (21.1)	7 (12.5)
Small for Gestational Age Infant	12 (9.3)	7 (9.3)	5 (9.3)
Birthweight (g)	3182 (637)	3289 (599)	3034 (664)
Male Neonatal Sex	72 (55.0)	41 (54.7)	31 (55.4)
*Worst HDP Diagnosis at Discharge from Delivery Hospitalization*			
None	36 (27.3)	33 (43.4)	3 (5.4)
Chronic Hypertension	21 (15.9)	9 (11.8)	12 (21.4)
Gestational Hypertension	35 (26.5)	16 (21.1)	19 (33.9)
Preeclampsia^*^	40 (30.3)	18 (23.7)	22 (39.3)
*Labs at Delivery Hospitalization*			
Highest Serum Cr	0.63 (0.55, 0.69)	0.61 (0.55, 0.69)	0.63 (0.55, 0.70)
Highest AST	20 (16, 26)	19 (15.5, 24)	20 (16, 27)
Lowest Platelet Count	209 (167, 241)	212 (167, 242)	209 (165, 241)
Urine Protein/Creatinine Ratio	0.2 (0.2, 0.4)	0.3 (0.2, 0.4)	0.2 (0.2, 0.3)
Abnormal Lab Values^a^ at Delivery Hospitalization	48 (36.4)	35 (46.1)	13 (23.2)
Antihypertensive Prescribed on Discharge	53 (40.2)	22 (29.0)	31 (55.4)
Oral Furosemide Prescribed on Discharge	25 (18.9)	10 (13.2)	15 (26.8)
Length of Delivery Hospitalization (days)	2 (2-3)	2 (2-3)	2 (2-4)

Data are N (%), mean (SD), median (25^th^, 75^th^ percentile).

*includes preeclampsia with and without severe features and superimposed preeclampsia.

^a^Defined as serum creatinine ≥1.1 mg/dL, platelets <100,000/uL, or AST ≥ 76 U/L (twice the upper limit of normal in our clinical lab).

*SBP, systolic blood pressure; DBP, diastolic blood pressure; BMI, body mass index; GA, gestational age; HDP, hypertensive disorders of pregnancy.*

### Severity Score at postpartum admission for hypertension management

Most patients had a Severity Score at PPA of one (77.3%), while only 2.3% had a Severity Score of three or higher (Table 2). All three patients with a Severity Score over two were in the normal BP group. A total of 71.1% of those with normal BP had a Severity Score of one compared to 85.7% of those with elevated BP, although there was no significant difference in Severity Scores between groups (adjusted β −0.11, 95% CI −0.33 to 0.11). In sensitivity analyses using the 150/100 mmHg or 130/80 mmHg cutoff, there were no differences in Severity Scores between groups (S1 and S2 Tables).

**Table 2:** Severity of PPA, days to PPA, and length of PPA by blood pressure at discharge.

	Total (n = 132)	Normal BP at Discharge from Delivery Hospitalization (<140/90 mmHg) (N = 76)	Elevated BP at Discharge from Delivery Hospitalization (SBP ≥ 140 or DBP ≥ 90 mmHg) (N = 56)	Estimate (95% CI)	Estimate (95% CI)^*^
Mean Severity Score at PPA	1.0 (0.6)	1.1 (0.7)	0.9 (0.4)	−0.12 (−0.33, 0.08)	−0.11 (−0.33, 0.11)
Frequency of Severity Scores					
0	17 (12.9)	11 (14.5)	6 (10.7)	NS	NS
1	102 (77.3)	54 (71.1)	48 (85.7)		
2	10 (7.6)	8 (10.5)	2 (3.6)		
3	2 (1.5)	2 (2.6)	0 (0.0)		
4	1 (0.8)	1 (1.3)	0 (0.0)		
Days to PPA	4 (2-7)	5 (3-8)	3 (1-5)	−1.67 (−3.16, −0.18)	−1.59 (−3.06, −0.12)
Length of PPA (days)	2 (2-3)	2 (2-3)	2 (2-3)	0.02 (−0.35, 0.39)	0.07 (−0.34, 0.48)

Data are mean (SD), median (25^th^, 75^th^ percentile), or N (%); linear regression used for days to PPA, length of PPA, and Severity Score and estimate is a beta coefficient.

*Adjusted for gestational age at delivery, diagnosis at discharge from delivery hospitalization, whether the patient was discharged on antihypertensives from delivery hospitalization, whether they were discharged on oral furosemide from delivery hospitalization, and length of delivery hospitalization. Model for Severity Score additionally adjusted for abnormal lab values at delivery hospitalization.

*PPA, postpartum admission; BP, blood pressure; CI, confidence interval; NS, not significant.*

### Days to postpartum admission for hypertension management

Overall, the median time to PPA for the entire cohort was 4 days (IQR 2–7). The median number of days to PPA was shorter in those with elevated BP: 3 days [IQR 1–5] versus 5 days [IQR 3–8] for those with normal BP, even after adjustment for confounders (β −1.59, 95% CI −3.06 to −0.12). The length of PPA was similar between groups (2 [IQR: 2–3] vs. 2 [IQR: 2–3] days) (Table 2). In the sensitivity analyses using the 130/80 mmHg cutoff or the 150/100 mmHg cutoff, there were no difference in days to PPA or length of PPA between groups (S1 and S2 Tables).

### Characterization of postpartum admission for hypertension management

**Fig 1:**
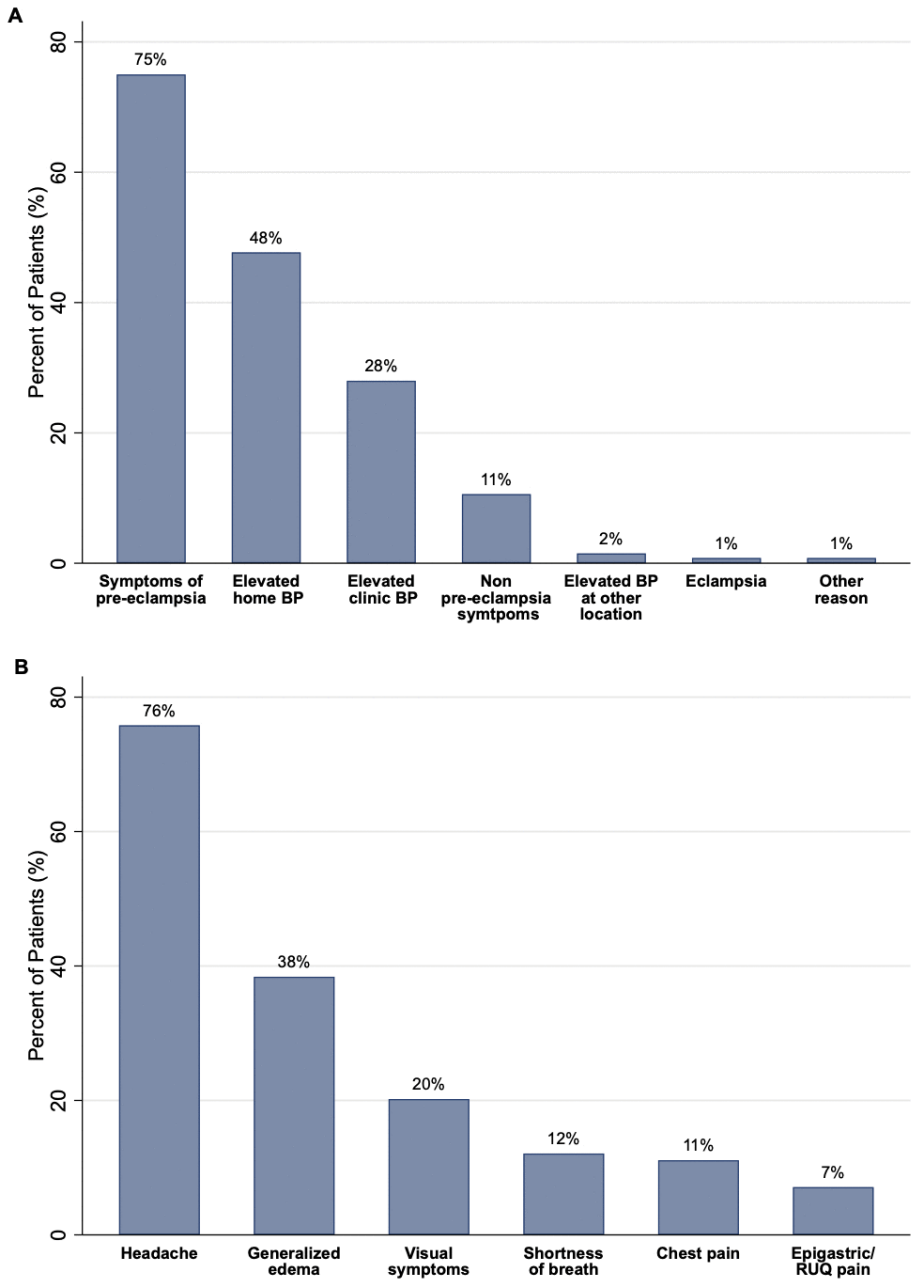
(A) Event in the postpartum period prompting evaluation and ultimately admission for management of hypertension. **(B)** Frequency of reported symptoms for patients reporting symptoms associated with HDP on presentation in the postpartum period (n = 99). Note that categories are not mutually exclusive for both A and B. Abbreviations: *HDP, hypertensive disorders of pregnancy; BP, blood pressure; RUQ, right upper quadrant.*

Most patients (>77%) presented with elevated BP at home or a clinical setting and 75% also reported symptoms of preeclampsia, with headache being the most common (75.8%) (Fig 1). The frequency of sustained severe range BP was high (86.2% overall; 83.8% in the normal BP group and 89.3% in the elevated BP group, p = 0.37). There were no cases of stroke or cardiac dysfunction at the time of the PPA, however, pulmonary edema was present in 4.6% (n = 6) of cases (5 in the normal BP group and 1 in the elevated BP group), and there was one case of PRES and eclampsia in the same patient (Table 3). The case of eclampsia presented on postpartum day 13 following a scheduled repeat Cesarean delivery at term that was not complicated by any HDP. As expected, continuation of medications at PPA was more common in the high BP group whereas new initiation of medications at PPA was more common in the normal BP group (Table 3).

**Table 3:** Interventions and complications during postpartum admission (PPA).

	Total (N = 132)	Normal BP at Discharge from Delivery Hospitalization <140/90 (N = 76)	Elevated BP at Discharge from Delivery Hospitalization (SBP ≥ 140 or DBP ≥ 90 mmHg) (N = 56)	p-value^d^
*Interventions at PPA*				
Continuation of oral antihypertensives	28 (21.2)	11 (14.5)	17 (30.4)	0.03
Initiation of oral antihypertensives	122 (92.4)	71 (93.4)	51 (91.1)	0.61
Administration of furosemide (oral or IV)	67 (50.8)	36 (47.4)	31 (55.4)	0.36
Administration of IV antihypertensives	61 (46.2)	34 (44.7)	27 (48.2)	0.69
Administration of IV magnesium sulfate	102 (77.3)	60 (79.0)	42 (75.0)	0.59
*Hypertensive Complications at PPA* ^*a*^				
Sustained severe range BPs^b^	112 (86.2)	62 (83.8)	50 (89.3)	0.37
Eclampsia	1 (0.8)	1 (1.3)	0 (0.0)	1.00
Pulmonary edema	6 (4.6)	5 (6.6)	1 (1.8)	0.24
PRES	1 (0.8)	1 (1.3)	0 (0.0)	1.00
Discharged on BP medications from PPA	130 (98.5)	74 (97.4)	56 (100.0)	0.51
No BP medications after delivery hospitalization to BP medications initiated during PPA	77 (58.3)	52 (68.4)	25 (44.6)	0.006
Worsening of diagnosis	101 (76.5)	67 (88.2)	34 (60.7)	<0.001
Abnormal lab values^c^ at PPA	12 (9.1)	11 (14.5)	1 (1.8)	0.01
Newly abnormal lab values^c^ at PPA	7 (5.3)	6 (7.9)	1 (1.8)	0.24

^a^As there were no cases of stroke or left ventricular dysfunction among patients with PPA, these are not listed in the table.

^b^Systolic BP ≥ 160 mmHg or diastolic BP ≥ 110 mmHg on at least two occasions more than 15 minutes apart.

^c^Defined as serum creatinine ≥1.1 mg/dL, platelets <100,000/uL, or AST ≥ 76 U/L (twice the upper limit of normal in our clinical lab).

^d^Chi-square tests and Fisher’s exact tests used for comparison between normal and elevated BP.

*PPA, postpartum admission; BP, blood pressure; PRES, posterior reversible encephalopathy syndrome*.

**Fig 2:**
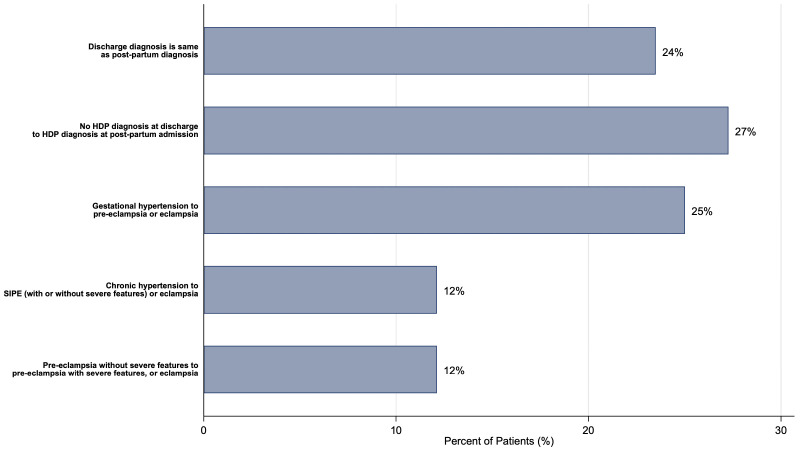
Frequencies of change in HDP diagnosis from discharge at delivery hospitalization to postpartum admission. Abbreviations: *SIPE, superimposed preeclampsia.*

Among individuals admitted with PPA, 24% had no change in their HDP diagnosis; 25% presented with preeclampsia after having GHTN; 12% presented with SIPE after having only CHTN at delivery hospitalization; and 12% presented with preeclampsia with severe features after having preeclampsia without severe features at delivery hospitalization. Interestingly, 27% (36 of 132) of the patients requiring PPA were newly diagnosed with HDP during the PPA (Fig 2). Unsurprisingly, patients with normal BP at discharge from delivery hospitalization more frequently had a change or worsening of their diagnosis (88.2% vs. 60.7%, p < 0.001) (Table 3). Abnormal laboratory values were more commonly present at delivery hospitalization (46.1% vs. 23.2%, p < 0.01) and at the time of PPA (14.5% vs. 1.8%, p = 0.01) in those with normal BP compared to those with elevated BP at discharge from delivery hospitalization, however, only a small number of patients had newly abnormal lab values at the time of PPA (Table 3).

Of the 36 patients with a *de novo* HDP diagnosis requiring PPA, the majority were diagnosed with preeclampsia with severe features (75.0%, n = 27), followed by gestational hypertension (16.7%, n = 6), preeclampsia without severe features (5.6%, n = 2), and eclampsia (2.8%, n = 1). 44.4% of these 36 patients were over the age of 35, 27.8% were nulliparous, 55.6% had a BMI ≥ 30 kg/m^2^, none had gestational or pregestational diabetes, and the median gestational age at delivery was 39.3 weeks. There were an additional 16 patients admitted for SIPE with or without severe features who, on discharge from delivery hospitalization, only had CHTN. When including these cases, 39.4% of PPAs in our cohort were for a new HDP diagnosis (other than CHTN).

Because the COVID-19 pandemic started during our study period, we determined if there were any differences in management before and after this time period, including length of stay at delivery hospitalization and PPA, rates of antihypertensive or furosemide prescription at discharge from delivery hospitalization or PPA, and interval from delivery to PPA. There were no differences among these parameters indicating that the COVID-19 pandemic did not impact our findings.

## Discussion

In this study of individuals requiring PPA, systolic BP ≥ 140 mmHg or diastolic BP ≥ 90 mmHg at discharge from delivery hospitalization was not associated with differences in the severity of HDP at the time of PPA. Higher BP at discharge from delivery hospitalization was associated with earlier presentation for admission. The frequency of severe presentations of HDP at PPA were similar between groups. Lastly, among patients requiring PPA for hypertension, up to 27% were for *de novo* HDP diagnoses.

Hypertensive complications are one of the leading causes for PPA [3]. Similar to other studies, we found that most PPAs occur prior to 7 days post-discharge, indicating that the traditional one-week follow-up visit may be inadequate at addressing postpartum BP changes [9,10]. However, what we uniquely found is that PPA for hypertension management is often for severe presentations that are not related to the severity of the antenatal HDP presentation. Despite having lower BP at delivery hospitalization, the low BP group frequently presented with severe disease (e.g., sustained severe range BPs, lab abnormalities). Additionally, the majority of postpartum pulmonary edema cases and the one eclampsia case were in the normal BP group. This suggests that lower BP targets may not completely mitigate the risk for postpartum worsening of HDP.

The 2013 ACOG guidelines recommend treatment in the postpartum period when BP is ≥ 150/100 mmHg [5], and the AHA considers stage 1 hypertension in non-pregnant adults when BP is ≥ 130/80 mmHg. Our sensitivity analyses in both situations did not detect any differences in HDP severity at PPA, although we may be underpowered to detect differences. A review of other national society guidelines demonstrates a lack of consensus and clarity regarding postpartum BP control. The National Institute for Health and Care Excellence (NICE) in the United Kingdom does not provide specific postpartum BP targets, but recommends treatment of hypertension antenatally when BPs are ≥ 140/90 mmHg, with a goal to lower them to ≤135/85 mmHg [14,15]. The Society of Obstetricians and Gynaecologists of Canada (SOGC) recommends treatment of severe hypertension (SBP ≥ 160 mmHg or DBP ≥ 110 mmHg) in the postpartum period, however, no other guidelines are provided regarding BP targets in the postpartum period [16]. The 2023 Society of Obstetric Medicine of Australia and New Zealand (SOMANZ) Hypertension in Pregnancy guidelines do not list a preferred BP target for the postpartum period [17]. This lack of recommendation by national societies reflects the limited data regarding postpartum BP targets to inform firm guidelines.

Our study also highlights the frequency at which PPAs are for a hypertensive complication newly presenting postpartum (*de novo* cases). Almost one third of our PPAs were for individuals without any HDP at the time of discharge from their delivery hospitalization. If we include cases of SIPE at PPA who had no preeclampsia concerns at the time of delivery hospitalization, this number increases to 39%. Although these are pregnancy-related complications, these are not traditional hospital re-admissions but rather necessary admissions for a *de novo* problem.

Further studies are needed to elucidate optimal BP targets to limit PPAs for hypertension. Studies that evaluated postpartum BP trajectories can help provide foundational information to develop intervention trials. One study that evaluated BP trajectories up to 42 days postpartum in individuals with CHTN, GHTN, and preeclampsia found that in all cases, postpartum BP peaked around day 5–7 postpartum, with rapid decline over the next week, followed by slower decline after 3 weeks [18]. This study, however, did not report BP trajectories stratified by those with HDP requiring PPA, nor did they separately quantify *de novo* HDP in the postpartum period.

As *de novo* postpartum HDP represents a unique phenomenon for which clinical indicators are not well known, early and frequent BP monitoring, such as that accomplished through postpartum remote monitoring programs (RPM) may be beneficial, particularly when they can be expanded to include not only those with an identified HDP at delivery hospitalization. Emerging studies suggest that RPM programs are likely cost-effective, including for reducing PPA [19,20]. Translational studies are urgently needed to understand this distinct population as these cases present days after placental delivery, raising the suspicion for persistent placental-associated factors affecting endothelial function.

We present a contemporary cohort with patient-level adjudication of HDP diagnoses at delivery hospitalization and admission in the postpartum period among those requiring PPA for hypertension. We add relevant data to the literature indicating that although BP ≥ 140/90 mmHg at delivery hospitalization is associated with earlier PPA compared to those with lower BP, severe disease at the time of PPA is frequent even in the low BP group. Secondly, we provide a contemporary estimate of PPAs for *de novo* postpartum HDP severe enough to warrant inpatient admission. We considered commonly used BP thresholds, aiding in clinically relevant interpretations of our findings. Our study is limited by its retrospective design with a primary focus on PPAs. As such, we are not able to comment on risk factors associated with PPA or how PPA can be predicted, although these have been previously extensively reported [9,10,21–23]. As our goal was to characterize admissions, we did not capture HDP cases, including both worsening or *de novo*, that were managed postpartum in the outpatient setting. Furthermore, our results may not be generalizable to settings that care for a different diversity of patients.

Severe HDP at the time of PPA for hypertension is common, regardless of BP control at discharge from delivery hospitalizations. Most PPAs for hypertensive complications occur before 7 days postpartum, regardless of BP control at delivery hospitalization, and those with higher BP (≥140/90 mmHg) presented earlier for PPA. HDP newly presenting postpartum and severe enough to warrant admission represents a substantial proportion of all PPAs, 27% in our cohort (up to 39% when including cases of CHTN that progressed to SIPE in the postpartum period). These findings warrant consideration of innovative strategies to limit and identify postpartum hypertensive complications, such as remote hypertension monitoring, and reconsideration of what is designated as an avoidable versus necessary admission for management of postpartum hypertension.

## Supporting information

S1 Table Severity of postpartum admission (PPA), days to PPA by blood pressure at discharge, using 130/80 mmHg as the cutoff. (DOCX)

S2 Table Severity of postpartum admission (PPA), days to PPA by blood pressure at discharge, using 150/100 mmHg as the cutoff. (DOCX)
