# Supplementary material for: Association between delivery hospitalization blood pressure and severity of postpartum admissions for hypertension
Source: PLoS One. 2026 Feb 18;21(2):e0342836. doi: 10.1371/journal.pone.0342836 (PMC12915911; doi:10.1371/journal.pone.0342836)
Supplement: S2 Table — (DOCX) [file pone.0342836.s002.docx]

| **Supplementary Table 2. Severity of postpartum admission (PPA), days to PPA by blood pressure at discharge, using 150/100 mmHg as the cutoff.** | | | | | |
| --- | --- | --- | --- | --- | --- |
|  | **Total (n=132)** | **Normal BP at Discharge from Delivery Hospitalization**  **(<150/100 mmHg)**  **(N=112)** | **Elevated BP at Discharge from Delivery Hospitalization**  **(SBP ≥150 or DBP ≥100 mmHg)**  **(N=50)** | **Estimate (95% CI)** | **Estimate (95% CI)^*^** |
| Severity Score at PPA | 1.0 (0.6) | 1.0 (0.6) | 0.9 (0.4) | -0.12 (-0.40, 0.16) | -0.03 (-0.31, 0.26) |
| 0 | 17 (12.9) | 14 (12.5) | 3 (15.0) |  |  |
| 1 | 102 (77.3) | 86 (76.8) | 16 (80.0) |  |  |
| 2 | 10 (7.6) | 9 (8.0) | 1 (5.0) |  |  |
| 3 | 2 (1.5) | 2 (1.8) | 0 (0.0) |  |  |
| 4 | 1 (0.8) | 1 (0.9) | 0 (0.0) |  |  |
| Days to PPA | 4 (2-7) | 4 (2-7) | 2.5 (1-5.5) | -1.32 (-3.40, 0.76) | -0.63 (-2.56, 1.30) |
| Length of PPA (days) | 2 (2-3) | 2 (2-3) | 2 (2-3) | -0.02 (-0.53, 0.50) | 0.07 (-0.45, 0.60) |
| Data are mean (SD), median (25^th^, 75^th^ percentile), or N (%); linear regression used for days to PPA, length of PPA, and Severity Score and estimate is a beta coefficient.  ^*^, Adjusted for gestational age at delivery, diagnosis at discharge from delivery hospitalization, whether the patient was discharged on antihypertensives from delivery hospitalization, whether they were discharged on oral furosemide from delivery hospitalization, and length of delivery hospitalization. Model for Severity Score additionally adjusted for abnormal lab values at delivery hospitalization.  *PPA, postpartum admission; BP, blood pressure; CI, confidence interval* | | | | | |
